# Chemically Modified DNAzyme with Enhanced Activity for Sensitive MicroRNA Imaging in Live Cells

**DOI:** 10.3390/molecules31081271

**Published:** 2026-04-12

**Authors:** Jiawen Chen, Juan Wang, Jiahuan Wang, Fulong Wang, Wenyu Cheng, Siqi Chen, Rui Mo, Hanyang Yu

**Affiliations:** 1 State Key Laboratory of Coordination Chemistry, College of Engineering and Applied Sciences, Chemistry and Biomedicine Innovation Center (ChemBIC), Nanjing University, Nanjing 210023, China; 502023340052@smail.nju.edu.cn (J.C.); wjhuan_27@163.com (J.W.); fulongwang0908@foxmail.com (F.W.); chengwenyu16@gmail.com (W.C.); siqichen0812@163.com (S.C.); 652023340040@smail.nju.edu.cn (R.M.); 2 State Key Laboratory of Coordination Chemistry, School of Chemistry and Chemical Engineering, Chemistry and Biomedicine Innovation Center (ChemBIC), ChemBioMed Interdisciplinary Research Center, Nanjing University, Nanjing 210023, China; dg20240105@smail.nju.edu.cn

**Keywords:** DNAzyme, chemically modified nucleic acid, miRNA detection and imaging

## Abstract

As critical regulators of gene expression, microRNAs (miRNAs) are key biomarkers and therapeutic targets in cancer. However, current methods for intracellular miRNA imaging are often limited by poor sensitivity and operational complexity. In this study, we identified a site-specifically modified DNAzyme variant, 11Bn, which exhibits up to 7-fold higher catalytic activity than the wild-type 8-17 through systematic screening. Using this variant, we constructed a DNAzyme-based sensor for miRNA-21 imaging in living cells. The sensor achieves a limit of detection of 7.89 nM, threefold lower than that of the wild-type sensor, and enables sensitive visualization of intracellular miRNA-21 without signal amplification. Moreover, it can capture dynamic changes in miRNA levels within cells, providing a versatile molecular tool for miRNA imaging and related biomedical applications.

## 1. Introduction

MicroRNAs (miRNAs) are short endogenous non-coding RNAs that regulate gene expression by mediating the degradation of messenger RNA (mRNA) or inhibiting its translation [1,2,3,4]. Aberrant miRNA expression is associated with various pathologies, such as cancer and metabolic diseases, positioning miRNAs as valuable tumor biomarkers and therapeutic targets [5,6,7]. For instance, miRNA-21 (miR-21) has been demonstrated to be upregulated in breast, lung, and colorectal cancers, whereas miRNA-143 exhibits significant downregulation [5]. Thus, identification and detection of miRNAs are not only crucial for elucidating cellular RNA functional networks [4], but also hold significant promise for enabling accurate and timely cancer diagnosis and therapy [8,9,10]. However, due to the low abundance, short length, and high family homology of miRNA, conventional detection methods, including reverse transcription polymerase chain reaction (RT-PCR) [11], Northern blotting [12] and in situ hybridization [13] (ISH), suffer from operational complexity, insufficient sensitivity and false-positive signals. These challenges underscore the urgent need for innovative tools enabling sensitive in situ miRNA imaging.

Catalytic DNAs, also known as deoxyribozymes (DNAzymes), have progressed in applications across fields such as gene therapy and biosensing [14,15,16] and serve as promising molecular tools for in situ miRNA detection within living cells. Nevertheless, the relatively low catalytic activity limits the practical application of DNAzymes. Current strategies to enhance DNAzyme activity typically rely on sophisticated preparation of chemically modified monomers, followed by solid-phase synthesis and activity screening [17,18,19]. This approach allows only one modified DNA to be prepared at a time, making the synthesis labor-intensive and time-consuming.

We have previously reported a simple and rapid chemoenzymatic method which enables site-specific installation of diverse artificial functional groups into DNAzymes [20]. Using this strategy in combination with activity screening, we identified a DNAzyme 10-23 variant bearing two site-specific modifications (CaBn) that exhibited the highest catalytic activity and enabled more sensitive imaging of intracellular magnesium ions. It was also observed that introducing a benzyl group at position 11 of DNAzyme 8-17 could enhance its catalytic activity. To demonstrate the broad applicability, we also tested the method on DNAzyme 8-17, and preliminary data showed that the introduction of a single site-specific chemical modification could enhance the activity of DNAzyme 8-17. Building on these findings, we herein performed a systematic and comprehensive site-specific chemical modification screening of DNAzyme 8-17 to identify and characterize activity-enhanced variants that can serve as new molecular tools for miRNA imaging.

By introducing artificial functional groups into the catalytic core of DNAzyme 8-17, we generated a total of 71 variants, including both singly and dually modified ones. Among them, the variant containing a single benzyl group at position 11 (11Bn) exhibits the highest activity enhancement, achieving a catalytic rate constant up to 7-fold higher than that of the unmodified DNAzyme. Based on this improved variant, we develop a multicomponent nucleic acid enzyme (MNAzyme) sensor for the specific detection of intracellular miR-21 [21]. The 11Bn-based sensor responds faster, generates stronger fluorescent signals and effectively distinguishes single-base mismatches in miR-21. Compared with the wild-type-based sensor, it offers a 3-fold lower limit of detection. When applied in living cells, the 11Bn-based sensor enables sensitive imaging of elevated miR-21 expression in cancer cells (MDA-MB-231) and can differentiate between varying levels of miR-21. This work offered a sensitive tool for sensitive miRNA imaging in living cells, with broad potential for DNAzyme-based applications in biological studies and clinical diagnostics.

## 2. Results

### 2.1. Chemical Modification Enhanced DNAzyme 8-17 Catalytic Activity

**Figure 1:**
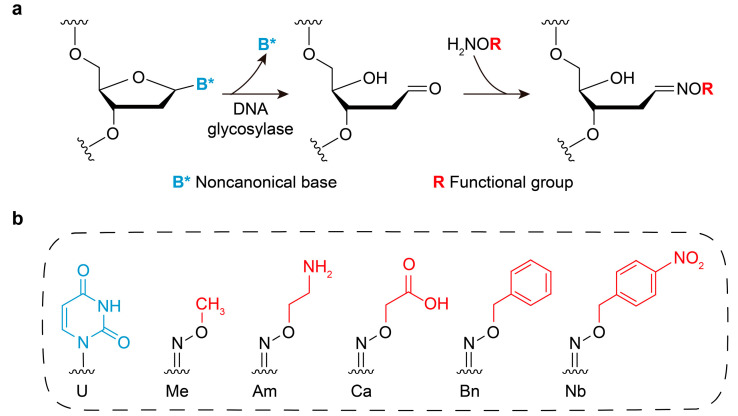
Site-specific chemoenzymatic modification strategy. (**a**) Chemoenzymatic introduction of functional groups (R) into DNA via glycosylase excision of noncanonical nucleobase (B*) followed by reaction with oxyamine compounds. (**b**) Structures of the noncanonical nucleobase (U, uracil) and functional groups used in this study.

The reported chemoenzymatic strategy is a two-step process involving noncanonical nucleobase excision and functional group installation [20]. Initially, uracil DNA glycosylase (UDG) is utilized to excise a single uracil (U) base within a DNA sequence, generating an abasic (AP) site [22]. Subsequently, various oxyamine compounds react with the resulting aldehyde group at the AP site to incorporate specific functional groups (Figure 1a). Five oxyamine compounds were chosen in this study for the introduction of methyl, amino, carboxyl, benzyl and nitrobenzyl groups (Figure 1b).

DNAzyme 8-17, a well-characterized catalytic DNA, is widely employed in biosensing and gene silencing [23,24,25]. Structurally, DNAzyme 8-17 comprises a catalytic core domain flanked by two substrate-binding arms. The catalytic core spans 15 nucleotides, featuring a stem-loop motif and a single-stranded region. All nine unpaired positions within the catalytic core (T1, A5, G6, C7, A11, C12, G13, A14, A15) were selected for the introduction of diverse chemical functionalities in order to evaluate the impact of each modification on DNAzyme’s catalytic activity [26].

In the modification process, the intermediate DNA containing an AP site is chemically unstable and highly susceptible to cleavage under alkaline conditions (200 mM NaOH, 37 °C, 15 min), which could be used for monitoring the removal of uracil nucleobase and installation of functional groups. For example, the DNAzyme variant A11U, where A at position 11 was replaced with U, remained intact under this alkaline condition. However, following UDG treatment (50 U/mL, 37 °C, 30 min), the resulting A11AP was efficiently cleaved into three fragments, confirming the presence of an AP site (Figure S1). After reaction of A11AP with methoxyamine (5 mM CH_3_ONH_2_, 37 °C, 30 min), the resulting A11Me variant again exhibited resistance to alkaline cleavage, indicating the successful introduction of a methyl group at position 11 (Figure S1).

**Figure 2:**
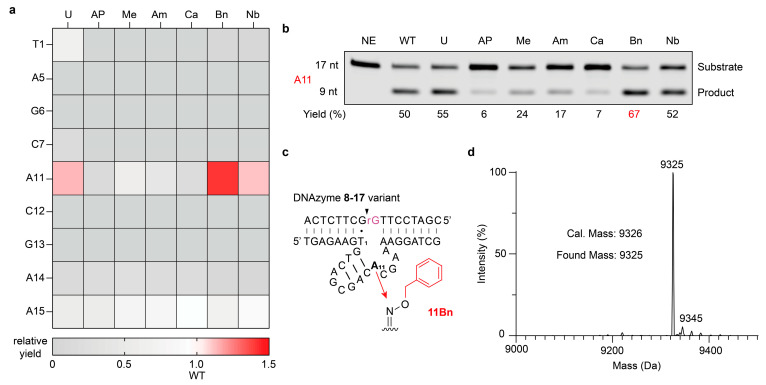
Site-specifically modified DNAzyme 8-17 shows improved catalytic activity. (**a**) Relative cleavage yields of chemically modified DNAzyme variants, normalized to that of the wild type (WT, set as 1). (**b**) Representative gel image showing the cleavage yields of DNAzyme 8-17 variants with distinct site-specific chemical modifications at position 11. Cleavage reactions were performed in 50 mM HEPES buffer (pH 7.5) containing 25 mM Mg^2+^ and 200 mM NaCl at 37 °C for 30 s. [Enzyme] = 500 nM. [Substrate] = 50 nM. (**c**) The sequence and structure of DNAzyme 8-17 variant 11Bn, in complex with the substrate. (**d**) Mass spectrum of chemically modified DNAzyme variant 11Bn.

Following chemical modification of nine positions, each with five oxyamine compounds, a total of 63 variants with single modification were produced, including those generated by U substitution and the AP-containing intermediates. We subsequently evaluated the catalytic activity of these variants under 25 mM Mg^2+^. Overall, most modifications substantially impaired the DNAzyme catalytic activity (Figure 2a). Among all the variants, the one with a benzyl modification at position 11 proved most effective, yielding 67% cleavage product compared with 50% for the wild type (WT) after 30 s of reaction (Figure 2b). Moreover, U substitution or nitrobenzyl modification at position 11 also led to moderate activity enhancement, yielding 55% and 52% cleavage product, respectively. Beyond position 11, variants modified at position 15 maintained partial activity as well, where only carboxyl- and nitrobenzyl-modified variants preserved activity levels comparable with the WT (Figure S2). All other variants exhibited either significantly diminished or undetectable cleavage activity. In particular, variants modified at conserved positions (A5, G6, C12, G13) showed no cleavage activity regardless of the specific substitution or modification applied, underscoring their functional indispensability (Figure S2). Among all, the variant with a benzyl modification at position 11 (11Bn) showed the highest improvement in catalytic activity (Figure 2c and Figure S2).

Building on the findings that modifications at position 11 enhance activity and at position 15 preserve activity, we next sought to evaluate the effect of simultaneously introducing two modifications at both positions on the DNAzyme catalytic activity. To construct such dually modified variants, two orthogonal “noncanonical nucleobase-DNA glycosylase” pairs were employed: hypoxanthine (Hx) with alkyladenine DNA glycosylase (AAG) and U with UDG (Figure S3a) [27,28]. Consistent with single-site modification, chemical cleavage analysis confirmed the successful incorporation of both modifications in the DNAzyme variants (Figure S3b). Among the dually modified variants, UNb (U at position 11; nitrobenzyl at 15) and BnNb (benzyl at position 11; nitrobenzyl at 15) variants exhibited enhanced cleavage activity, yielding 48% and 53% cleavage product, respectively, versus 41% for WT. In contrast, variants containing an AP site at either position displayed markedly reduced activity. The remaining variants retained substantial cleavage activity (Figure S3c). We therefore selected the BnNb variant, which showed the highest cleavage yield, for further kinetic characterization in comparison with the singly modified 11Bn (Figure S4). The results indicated that under the 1 mM Mg^2+^ condition, the observed rate constant (*k*_obs_) of BnNb was slightly lower than that of 11Bn (2.13 h^−1^ vs. 2.26 h^−1^) (Figure S4). While BnNb exhibited enhanced cleavage activity over WT, it did not outperform the singly modified 11Bn variant.

Collectively, based on the systematic screening of singly and dually modified DNAzyme variants, 11Bn emerged as the one with the highest catalytic yield (Figure 2c). Mass spectrometry analysis further confirmed successful introduction of the benzyl functional group (Figure 2d and Figure S5).

**Figure 3:**
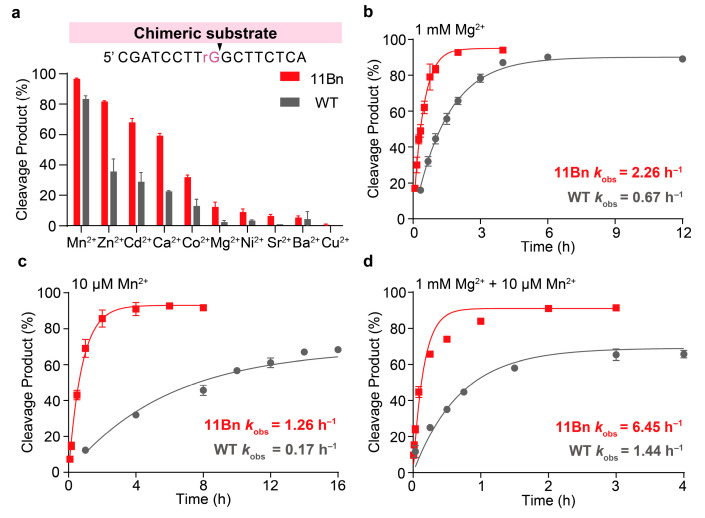
Biochemical characterization of the modified DNAzyme 11Bn-catalyzed cleavage of chimeric RNA substrate. (**a**) Divalent metal ion dependence of WT and 11Bn-catalyzed cleavage reaction of DNA–RNA chimeric substrate. Cleavage reactions were performed in 50 mM HEPES buffer (pH 7.5) containing 200 mM NaCl supplemented with 1 mM various divalent metal ions at 37 °C for 5 min. (**b**–**d**) *k*_obs_ values of WT- and 11Bn-catalyzed cleavage reaction of DNA–RNA chimeric substrate performed in 50 mM HEPES buffer (pH 7.5) containing 200 mM NaCl and different metal ion cofactors (1 mM Mg^2+^, 10 μM Mn^2+^ or both, respectively) at 37 °C. [Enzyme] = 500 nM. [Substrate] = 50 nM. The error bars denote ± S.D. of the mean for *n* = 3 independent replicates.

To gain insight into this enhanced variant, we next performed detailed biochemical characterization of 11Bn. Given that the cleavage activity of DNAzyme 8-17 can be supported by various divalent metal ions [29,30], we evaluated the performance of both WT DNAzyme and the 11Bn variant in cleaving DNA–RNA chimeric substrate across different metal ion cofactors. This aimed to assess the impact of the benzyl modification on its cleavage activity as well as metal ion responsiveness. In the presence of 1 mM metal ion, 11Bn consistently demonstrated higher catalytic activity than the WT, as evidenced by increased cleavage yields (Figure 3a). Additionally, both exhibited nearly identical metal ion preference, roughly in the order of Mn^2+^ > Zn^2+^ > Cd^2+^ > Ca^2+^.

The kinetic characterization of 11Bn demonstrated a notable enhancement of *k*_obs_ over WT under simulated physiological conditions. Specifically, in the presence of 1 mM Mg^2+^, 11Bn exhibited a 3.4-fold increase in *k*_obs_ (2.26 h^−1^ vs. 0.67 h^−1^; Figure 3b). Under a 10 μM Mn^2+^ condition, the enhancement was even more pronounced, with 11Bn achieving a 7.4-fold higher *k*_obs_ (1.26 h^−1^ vs. 0.17 h^−1^; Figure 3c). Catalytic rates were further determined under conditions with both metal ions present. In this dual-ion system, 11Bn maintained a 4.5-fold catalytic rate advantage over WT (6.45 h^−1^ vs. 1.44 h^−1^; Figure 3d). This rate enhancement was similarly observed across a range of varying Mg^2+^ or Mn^2+^ concentrations, with the data summarized in Table S2. A Michaelis-Menten kinetic analysis under multiple turnover conditions revealed a catalytic turnover number (*k*_cat_) of 11.6 h^−1^ and a Michaelis constant (*K*_M_) of 4.3 μM for 11Bn (Figure S6).

**Figure 4:**
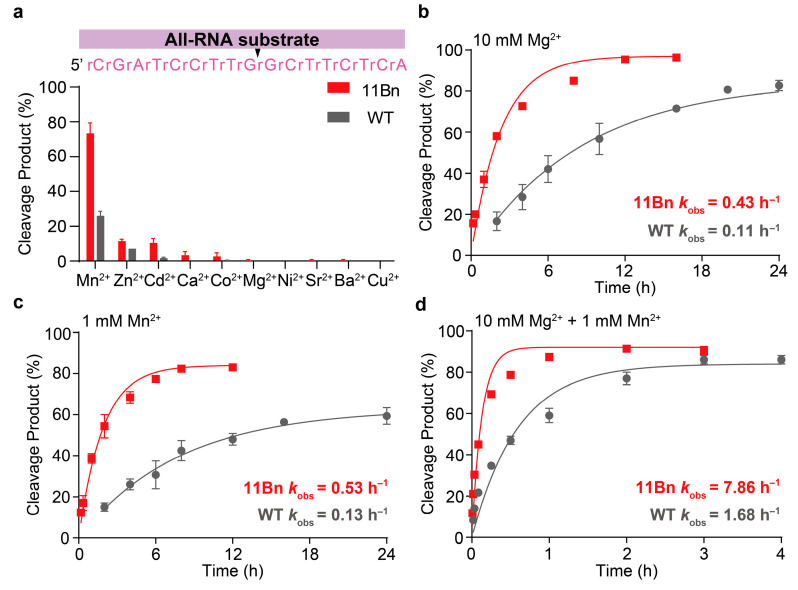
Biochemical characterization of the modified DNAzyme 11Bn-catalyzed cleavage of all RNA substrate. (**a**) Divalent metal ion dependence of WT- and 11Bn-catalyzed cleavage reaction of all-RNA substrate. Cleavage reactions were performed in 50 mM HEPES buffer (pH 7.5) containing 200 mM NaCl supplemented with 1 mM various divalent metal ions at 37 °C for 4 h. (**b**–**d**) *k*_obs_ values of WT- or 11Bn-catalyzed cleavage reaction of all-RNA substrate performed in 50 mM HEPES buffer (pH 7.5) containing 200 mM NaCl and different metal ion cofactors (10 mM Mg^2+^, 1 mM Mn^2+^ or both, respectively) at 37 °C. [Enzyme] = 500 nM. [Substrate] = 50 nM. The error bars denote ± S.D. of the mean for *n* = 3 independent replicates.

We next evaluated the catalytic performances of both 11Bn and WT DNAzymes on all-RNA substrates. Again, the chemical modification did not alter the metal ion preference of the DNAzyme, as both variants displayed an identical order of preference (Figure 4a). Generally, cleavage rates for all-RNA substrates were lower than those for chimeric substrates. Nevertheless, 11Bn consistently exhibited significantly higher catalytic rates than WT across all tested ion conditions. Specifically, 11Bn showed a 3.9-fold increase in *k*_obs_ under 10 mM Mg^2+^ (0.43 h^−1^ vs. 0.11 h^−1^), a 4.1-fold increase under 1 mM Mn^2+^ (0.53 h^−1^ vs. 0.13 h^−1^) and a 4.7-fold increase under dual-metal conditions (7.86 h^−1^ vs. 1.68 h^−1^) (Figure 4b–d). To sum up, the chemical modification did not alter the metal ion preference of DNAzyme, and under all tested conditions, 11Bn consistently demonstrated higher catalytic activity than WT.

### 2.2. DNAzyme 11Bn-Based Biosensor of microRNA

**Figure 5:**
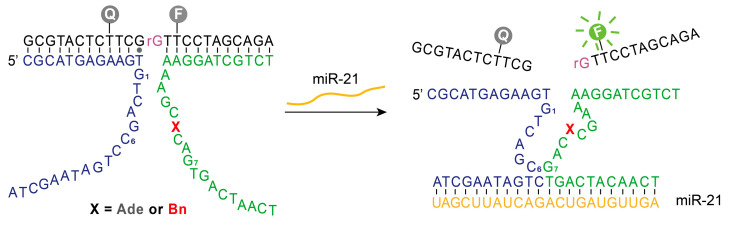
Design of MNAzyme (multicomponent nucleic acid enzyme)-based sensor for microRNA detection. Recognition of target microRNA-21 (miR-21) by MNAzyme triggers enzymatic cleavage of the fluorophore-labeled substrate, generating detectable fluorescent signals. F: 6-carboxyfluorescein. Q: BHQ-1 quencher. Green: Fragment 1; blue: Fragment 2; black: chimeric substrate; yellow: target miR-21.

Given the observed higher catalytic activity of 11Bn, we reasoned that this variant could enable more sensitive biomolecular detection. We therefore designed a microRNA biosensor based on DNAzyme 11Bn by employing a multicomponent nucleic acid enzyme (MNAzyme) system [21]. This system comprises two split DNAzyme fragments and a substrate strand labeled with a fluorophore–quencher pair. Each split fragment is extended with a sequence complementary to the target microRNA. In the absence of the target, the two fragments cannot self-assemble into a functional DNAzyme. Upon binding of the microRNA trigger, the fragments are brought together, restoring catalytic activity and cleaving the substrate, which separates the fluorophore from the quencher and generates a fluorescent signal. In essence, this MNAzyme design translates the presence of a specific microRNA into a fluorescent readout (Figure 5).

**Figure 6:**
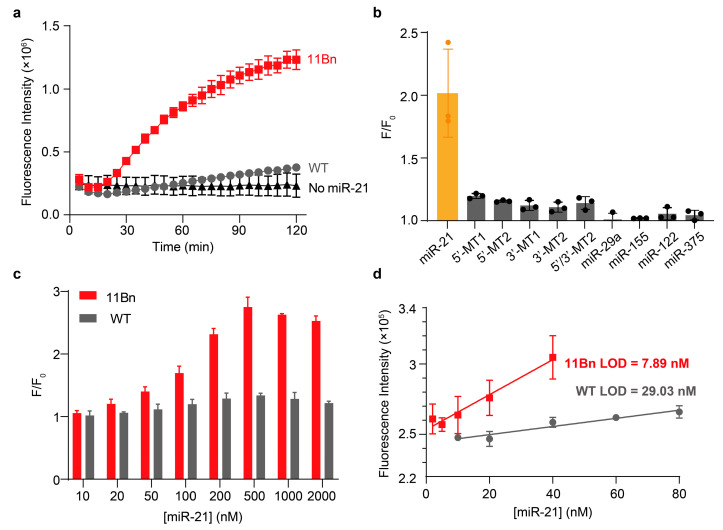
Characterization of MNAzyme-based sensor for microRNA detection. (**a**) Time-dependent fluorescence intensity of 11Bn- or WT-based MNAzyme. The reactions were performed in 50 mM HEPES buffer (pH 7.5) containing 10 mM Mg^2+^ and 200 mM NaCl at 37 °C for 2 h. [Enzyme] = 200 nM. [Substrate] = 200 nM. (**b**) Specificity of the 11Bn-based MNAzyme for miRNA detection. [Target] = 200 nM. MT1 and MT2: single- and double-base mismatch mutants of miR-21. The prefix (3′ or 5′) denotes the mismatch position. F and F_0_ represent fluorescence intensities in the presence and absence of the target, respectively. (**c**) MiR-21 concentration-dependent fluorescence intensity of 11Bn/WT-based MNAzyme. (**d**) Linear region of miR-21 concentration-dependent fluorescence increase. The limit of detection was calculated as 3 × σ/slope. Reactions were performed in 50 mM HEPES buffer (pH 7.5) containing 1 mM Mg^2+^ and 200 mM NaCl at 37 °C for 4 h. [Enzyme] = 200 nM. [Substrate] = 400 nM. The error bars in (**a**–**d**) denote ± S.D. of the mean for *n* = 3 independent replicates.

We selected microRNA-21 (miR-21) as the detection target due to its well-documented upregulation in various cancers. Two systems were designed, differing in the lengths of the substrate strand and its corresponding DNAzyme binding arms. Fluorescence assays revealed that, within 2 h of incubation with miR-21, the system with short binding arms (8 nt and 7 nt) showed no significant increase in fluorescence signal (Figure S7). In contrast, the system with longer binding arms (both 11 nt) produced a clear fluorescence signal above background (Figure 6a) [31,32,33]. Notably, the 11Bn-based MNAzyme exhibited markedly stronger fluorescent signals as early as 30 min compared with the no-target control, while the signal from the WT-based MNAzyme remained barely discernible from the background even at 120 min.

We optimized the stoichiometry by varying the substrate equivalent from 1 to 10 while keeping miR-21 and enzyme at 1:1. The highest F/F_0_ was observed with two equivalents of substrate, indicating that one MNAzyme can process multiple substrate molecules. Further increasing the substrate to 5 and 10 equivalents led to a decline in F/F_0_ due to excessive background fluorescence (Figure S8). The multi-turnover activity was confirmed by gel-based cleavage assays using a fluorophore-labeled, quencher-free substrate, where near-complete substrate cleavage was achieved even at 10-fold substrate excess (Figure S9). However, when enzyme was increased to 2 or 5 equivalents while keeping miR-21 constant, no kinetic gain was observed, as excess enzyme fragments cannot assemble without additional miR-21 trigger. Based on these results, we selected miR-21:enzyme:substrate = 1:1:2 as the optimized ratio.

We next evaluated the specificity of the MNAzyme system by testing its response to five mutated miR-21 sequences: 3′-MT1 and 3′-MT2 (single- and double-base mismatch near the 3′ end, respectively), 5′-MT1 and 5′-MT2 (mismatch near the 5′ end) and 5′/3′-MT2 (one mismatch near each end). Four other non-target miRNAs (miR-29a, miR-155, miR-122 and miR-375) were also tested. The results showed that only miR-21 triggered a pronounced fluorescence increase, while none of the interfering sequences produced a significant fluorescent signal (Figure 6b). These findings demonstrate that the 11Bn-based MNAzyme system exhibits high selectivity for its target miRNA.

We then assessed the sensitivity of the 11Bn- and WT-based MNAzymes by measuring their response to miR-21 of varying concentrations. The results showed that at 500 nM miR-21, the 11Bn MNAzyme reached a maximal F/F_0_ value of 2.75, approximately twice that of the WT MNAzyme (Figure 6c). The limit of detection (LOD), calculated as 3 × σ/slope with the fluorescence intensity (F), was 7.89 nM for the 11Bn-based MNAzyme, about three times lower than that of the WT-based system (Figure 6d). These results demonstrate that the chemical modification enhances the detection sensitivity of the MNAzyme system for miRNAs.

To enable intracellular miRNA detection and imaging, we developed a hybrid sensor system by integrating the MNAzyme with MnO_2_ nanosheets (MnO_2_ NS). The MnO_2_ NS serves a dual role. First, as a delivery carrier, it adsorbs the MNAzyme components via van der Waals interactions, thereby enhancing their cellular uptake through endocytosis. Second, after internalization, the MnO_2_ NS is reduced by intracellular glutathione (GSH), releasing Mn^2+^ and causing the dissociation of the nucleic acids [34,35]. The released components then self-assemble in situ into active MNAzyme in the presence of intracellular miR-21. The liberated Mn^2+^, together with endogenous Mg^2+^, facilitates substrate cleavage, thereby generating a quantifiable fluorescence signal.

**Figure 7:**
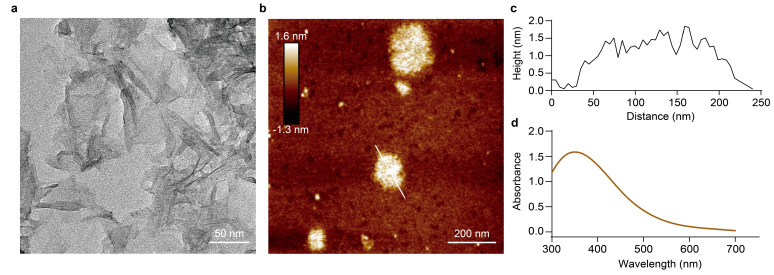
Characterization of MnO_2_ nanosheet (NS). (**a**) TEM and (**b**) AFM images of MnO_2_ NS. (**c**) Height profile along the white line shown in Figure 4b. (**d**) UV–vis absorption spectrum of MnO_2_ NS.

Synthesized MnO_2_ NS was characterized by transmission electron microscopy (TEM), revealing a uniform sheet-like morphology (Figure 7a). Atomic force microscopy (AFM) further indicated a two-dimensional structure with an average height of ~1.5 nm and lateral dimensions of ~200 nm (Figure 7b,c). Additionally, the UV–visible absorption spectrum displayed a prominent peak at 370 nm, consistent with the optical property of MnO_2_ NS (Figure 7d) [36]. Collectively, these results confirm the successful synthesis of MnO_2_ NS.

**Figure 8:**
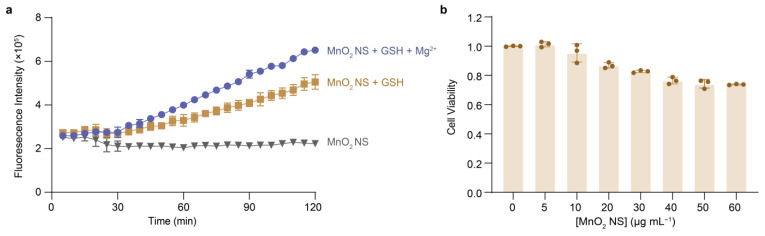
Functional evaluation of MnO_2_ NS for MNAzyme activation and biocompatibility. (**a**) Time-dependent fluorescence intensity of 11Bn-based MNAzyme at 520 nm under distinct conditions. The reactions were performed in 50 mM HEPES buffer (pH 7.5) containing MnO_2_ NS, 200 mM NaCl (gray); 0.2 mM GSH, MnO_2_ NS, 200 mM NaCl (brown); or 1mM Mg^2+^, 0.2 mM GSH, MnO_2_ NS, 200 mM NaCl (indigo) at 37 °C for 2 h, respectively. [Enzyme] = 200 nM. [Substrate] = 400 nM. (**b**) Cytotoxicity assay of MnO_2_ NS of different concentrations incubated with MDA-MB-231 cells for 24 h. The error bars in (**a**,**b**) denote ± S.D. of the mean for *n* = 3 independent replicates.

To evaluate the potential of MnO_2_ NS in MNAzyme-based intracellular miRNA sensing, we first examined its reduction behavior in the presence of glutathione (GSH) of varying concentrations. The results showed a concentration-dependent decrease in the characteristic UV–vis absorption peak of MnO_2_ NS at 370 nm, indicating effective GSH-mediated reduction and degradation of nanosheet (Figure S10). We then investigated the activation of the MNAzyme under different conditions. While unreduced MnO_2_ NS alone failed to activate the MNAzyme, the addition of GSH led to a marked increase in fluorescence output, demonstrating that the reduction of MnO_2_ NS effectively triggers DNAzyme-mediated cleavage (Figure 8a). Finally, in the presence of both GSH and Mg^2+^, the system produced a faster and more pronounced fluorescence enhancement, consistent with the synergistic activation of the DNAzyme by Mn^2+^ and Mg^2+^ observed earlier (Figure 3).

Before assessing the potential of this sensor for intracellular miRNA detection and imaging, we evaluated the cytotoxicity of the nanosheets using a CCK-8 assay. MnO_2_ NS of varying concentrations (0–60 μg/mL), as determined by a standard curve (Figure S11), were incubated with MDA-MB-231 cells for 24 h. The results showed that even at the highest concentration of 60 μg/mL, cell viability remained approximately 70% (Figure 8b). This low cytotoxicity, coupled with efficient intracellular activation by GSH, demonstrates the feasibility of using MnO_2_ NS for live-cell applications.

### 2.3. Sensitive Imaging of Intracellular microRNA

To evaluate the cellular uptake and intracellular performance of the sensor, we tested two cell lines: MCF10A, a non-malignant human breast epithelial cell line with low miR-21 expression, and MDA-MB-231, a human breast cancer cell line with elevated miR-21 expression (Figure S12) [37,38]. In MDA-MB-231 cells, incubation with MNAzyme system alone (without MnO_2_ NS) for 6 h produced no detectable fluorescence under confocal microscopy, whereas co-incubation with MnO_2_ NS led to prominent cytoplasmic fluorescence. In contrast, MCF10A cells treated with both the MNAzyme and MnO_2_ NS exhibited much weaker fluorescent signals (Figure S12). These results demonstrate that MnO_2_ NS effectively facilitates the cellular delivery of the MNAzyme-based sensor without requiring transfection reagents. Moreover, the clear difference in fluorescence intensity between MDA-MB-231 and MCF10A cells reflects the sensor’s ability to distinguish elevated miR-21 expression in cancer cells from normal cells, highlighting its potential for cancer cell detection.

**Figure 9:**
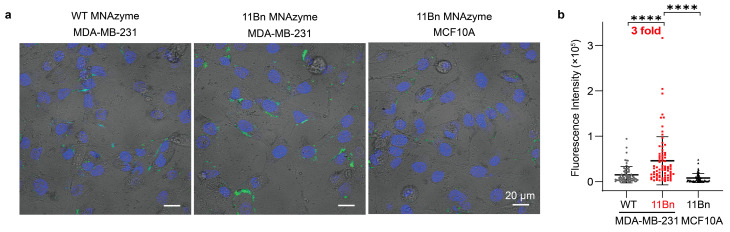
Sensitive imaging of intracellular miRNA. (**a**) Confocal fluorescence images and (**b**) quantification of MDA-MB-231 and MCF10A cells after incubation with WT- or 11Bn-based MNAzymes and MnO_2_ NS (33 μg/mL) for 4 h. Statistical analysis was performed using a two-tailed *t*-test. ****: *p* < 0.0001. Data represent mean ± S.D. (*n* = 71, 78 and 71 cells for WT-MDA-MB-231, 11Bn-MDA-MB-231 and 11Bn-MCF10A, with fluorescence intensities of 1.5 × 10^4^, 4.6 × 10^4^ and 7.9 × 10^3^, respectively).

To compare the ability of the 11Bn- and WT- based sensors to distinguish cancer cells from normal cells, we incubated the sensors with cell lines expressing miR-21 of different endogenous levels. The confocal microscopy results showed that the 11Bn sensor effectively distinguished cancer cells from normal cells, producing strong fluorescence only in miR-21-overexpressing MDA-MB-231 cells, with minimal signal in MCF10A cells. Moreover, the 11Bn sensor produced significantly stronger fluorescent signals than the WT sensor, with average intensities of 4.6 × 10^4^ and 1.5 × 10^4^, respectively, representing a threefold increase (Figure 9a,b). Flow cytometry analysis of MDA-MB-231 cells further confirmed the superior performance of the 11Bn-based sensor (Figure S13).

We further evaluated both sensors in the human lung adenocarcinoma A549 cell line [38]. Again, the 11Bn sensor showed enhanced sensitivity, generating clear fluorescence, while the WT sensor yielded insufficient signal for reliable miR-21 detection (Figure S14). The average fluorescence intensity of the 11Bn sensor was approximately four times higher than that of the WT sensor. Flow cytometric analysis of A549 cells also supported this observation, showing that the 11Bn sensor yielded significantly stronger fluorescence signals than the WT sensor (Figure S15). These results confirm that the 11Bn sensor is more sensitive than the WT sensor in detecting and imaging miR-21, and it can effectively differentiate cancer cells from normal cells, demonstrating its broad applicability in cancer cell imaging.

**Figure 10:**
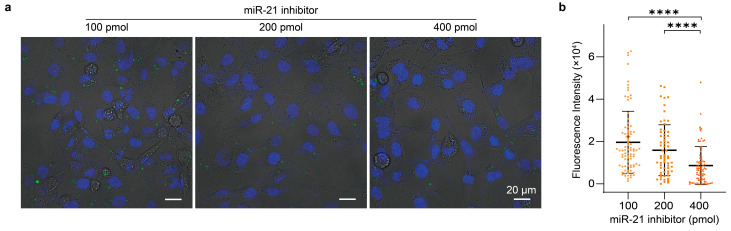
Monitoring dynamic changes of intracellular miRNA. (**a**) Confocal fluorescence images and (**b**) quantification of MDA-MB-231 cells, first treated with a miRNA inhibitor of different amounts (100, 200, 400 pmol) for 24 h and then incubated with 11Bn-based MNAzyme-MnO_2_ NS sensors for 4 h. Statistical analysis was performed using a two-tailed *t*-test. ****: *p* < 0.0001. Data represent mean ± S.D. (*n* = 86, 64 and 77 cells for 100 pmol, 200 pmol and 400 pmol, with fluorescence intensities of 2.0 × 10^4^, 1.6 × 10^4^ and 8.6 × 10^3^, respectively).

Finally, we demonstrated the ability of the sensor to monitor changes in intracellular miR-21 levels. MDA-MB-231 cells were transfected with an miR-21 inhibitor of increasing amounts (100, 200 and 400 pmol). The efficacy of the inhibitor was validated by qPCR (Figure S16). After treatment, a corresponding decrease in fluorescence signals was observed, with average intensities of 2.0 × 10^4^, 1.7 × 10^4^ and 8.6 × 10^3^, respectively (Figure 10a,b). These results show that the 11Bn sensor can reliably track dynamic changes in intracellular miR-21 levels and support its potential for semi-quantitative detection in complex biological environments.

## 3. Discussion

The chemoenzymatic strategy offers a more streamlined alternative to the conventional synthesis of modified DNA via preparation of nucleoside phosphoramidites followed by solid-phase synthesis, enabling rapid and parallel generation of multiple DNAzyme variants with site-specific modifications [20]. Inspired by our previous work, we extended this strategy to DNAzyme 8-17 and performed a systematic modification screening, leading to the identification of 11Bn as the variant with the highest activity enhancement and further illustrating the utility of this method across different DNAzyme frameworks. While the extent of activity enhancement varies among different frameworks, the 7.4-fold improvement of 11Bn represents a notable achievement among other reported chemically modified variants of DNAzyme 8-17 [39,40,41,42]. Moreover, the detailed biochemical characterization of 11Bn, including its enhanced activity under dual-metal ion conditions, provides a useful reference for the further application of this molecular tool.

In addition, our comprehensive screening across different positions of the catalytic core provides new experimental insights into the structure–function studies of 8-17. The chemical modification results are consistent with the established consensus of DNAzyme 8-17 that the conserved stem-loop (positions 2–10) and the downstream “CGAA” motif (positions 12–15) are intolerant to alteration [23], as modifications at these sites largely abolished activity. In contrast, position 11 is known to be mutationally permissive [26,39,43,44,45]. Confirming this, we found that substitution of A11 with uracil maintained activity, while installation of a benzyl group significantly enhanced catalysis, yielding a reaction rate up to 7.4-fold higher than the wild type. This demonstrates that moving beyond canonical nucleobases or analogs to incorporate tailored chemical moieties can markedly improve DNAzyme performance, a key advance enabled by our chemoenzymatic methodology.

Structural studies of the 8-17 DNAzyme reveal that the nucleobase at position 11 does not form hydrogen bonds or base pairs but instead engages in stacking interactions with an adjacent G:C pair, likely contributing to structural stability [46,47]. This explains the frequent tolerance of mutations at this site. In our work, installing a benzyl group, which similarly lacks hydrogen-bonding capacity, did not impair activity but enhanced it. We speculate that the benzyl moiety may reinforce these crucial stacking interactions, thereby improving the structural integrity of the catalytic core and boosting activity.

Structural models also place the nucleotide at position 11 near a metal ion binding pocket, suggesting that modifications here could potentially alter metal ion responsiveness. Previous studies have shown that introducing bases with distinct chemical properties (e.g., hypoxanthine or an abasic site) can shift catalytic efficiency differentially across ions [26,44], for example reducing activity in Mg^2+^ but increasing it in Ca^2+^, highlighting how the chemical nature of the modification influences ion-dependent activity. Conversely, we find that the A11Bn variant retains the same metal ion preference as the wild type, which aligns with reports that certain substitutions (e.g., A11T) do not reshape selectivity [39], likely due to the absence of metal-coordinating groups on the benzyl ring. Taken together, these results demonstrate that judicious chemical functionalization can both enhance catalysis and offer a means to modulate the innate metal ion selectivity of the DNAzyme.

As a well-established cancer biomarker, miR-21 plays a critical role in tumor proliferation, migration, and apoptosis inhibition. Its aberrant expression is associated with multiple cancer types, making its detection and imaging essential for both fundamental research and clinical applications [2,48]. Current detection strategies [49,50], such as those based on CRISPR/Cas [51] or SplintR ligase [52], rely on exogenous proteins and thus face inherent challenges including biological instability, immunogenicity and delivery difficulties. While cascade amplification systems like hybridization chain reaction (HCR) [50] or asymmetric CRISPR circuits [53] can enhance signal intensity, their exponential amplification often leads to background leakage and non-linear biases. Conventional imaging methods such as fluorescence in situ hybridization (FISH) [54,55,56] require cell fixation and are therefore unsuitable for dynamic live-cell studies.

Our chemically modified DNAzyme-based sensor offers a simpler and more accessible tool for miRNA imaging. The sensor is easy to prepare, and the use of MnO_2_ nanosheets facilitates both cellular uptake and DNAzyme activation. Leveraging the enhanced catalytic activity from chemical modification and the multi-turnover nature of the DNAzyme, we achieved a detection limit of 7.89 nM without additional signal amplification. Overall, this platform provides high-specificity live-cell imaging of dynamic intracellular nucleic acid changes, enabling semi-quantitative analysis of relative miRNA levels, which positions it as a promising tool for early cancer diagnosis and prognostic monitoring. Meanwhile, achieving quantification in living cells remains a meaningful direction for future research and will be the focus of our subsequent studies.

## 4. Materials and Methods

### 4.1. Mass Spectrometry

High-resolution mass spectra (HRMS) were recorded on a Thermo Fisher Q-Exactive Orbitrap Mass Spectrometer (Waltham, MA, USA) and Bruker UltrafleXtreme MALDI-TOF Mass Spectrometer (Billerica, MA, USA).

### 4.2. Oligonucleotides

All-RNA substrate was purchased from Biosyntech (Suzhou, China), and the remaining oligonucleotides were purchased from Sangon Biotech (Shanghai, China). DNAzyme 8-17 used for single site-specific functionalization contains uracil at the specified location (Table S1). DNAzyme 8-17 used for dual modifications contains uracil and hypoxanthine at position 11 and 15 of the catalytic core, respectively. The DNA–RNA chimeric substrates used in cleavage reaction contain a Cy5.5 modification on the 5′ end and one internal RNA residue (rG) (Table S1). The all-RNA substrate contains a Cy5.5 modification on its 5′ end. The DNA–RNA chimeric substrate for MNAzyme in miR-21 imaging contains a FAM (6-carboxy-fluorescein) modification on position 11, a BHQ-1 (black hole quencher-1) on position 16 and one internal RNA residue (rG).

### 4.3. Oxyamine Compounds

O-Methylhydroxylamine hydrochloride was purchased from Shanghai Yuanye Biotechnology (Shanghai, China). O-(4-Nitrobenzyl)hydroxylamine hydrochloride was purchased from Shanghai Bepharm Science & Technology (Shanghai, China). 2-(Aminooxy)ethanamine dihydrochloride was purchased from Shanghai Acmec Biochemical (Shanghai, China). O-(Carboxymethyl)hydroxylamine hemihydrochloride was purchased from Shanghai 9dingchem (Shanghai, China). O-Benzylhydroxylamine hydrochloride was purchased from Shanghai Macklin Biochemical (Shanghai, China).

### 4.4. Site-Specific Modification of DNA

To install a single chemical modification to the specified position, the corresponding DNA containing a uracil base (Table S1) was first treated with uracil DNA glycosylase (UDG, New England Biolabs, Ipswich, MA, USA) for uracil excision. The reaction mixture contained 10 μM DNA and 50 U/mL UDG in 20 mM Tris buffer (pH 8.0) containing 1 mM DTT and 1 mM EDTA and was incubated at 37 °C for 30 min [57]. After base excision, the AP site-containing DNA sequence was purified by ethanol precipitation. Then, 10 μM AP site-containing DNA sequence was incubated with 5 mM oxyamine compound in 50 mM HEPES (pH 7.0) containing 100 mM NaCl at 37 °C for 30 min [20]. Finally, the chemically modified DNA was purified by ethanol precipitation or on a desalting column (Bio-Rad, Hercules, CA, USA).

To install dual-chemical modifications to the specified positions, the DNA sequence with uracil and hypoxanthine bases was first annealed to a biotinylated complementary sequence (Table S1) at a ratio of 1:1.2 (94 °C, 5 min; 4 °C, 5 min). Then the double-stranded DNA (dsDNA) was treated with human alkyladenine DNA glycosylase (AAG, New England Biolabs) for hypoxanthine excision. The reaction was performed under 2 μM DNA, 200 U/mL AAG, 50 mM acetate, 1 mM DTT, 1 mM EDTA, 100 mM NaCl, and 0.1 mg/mL BSA, pH 5.8, 37 °C for 4 h [58]. After base excision, the sequence was purified by ethanol precipitation. The AP site-containing dsDNA was incubated with O-benzylhydroxylamine under the oxyamine reaction condition. The modified dsDNA was immobilized onto a streptavidin agarose resin column (Thermo Fisher, Waltham, MA, USA), and then the modified strand was eluted by 200 mM NaOH and purified by ethanol precipitation or on a desalting column. Such modified DNA contained a benzyl modification and a uracil base and was further modified using a UDG/oxyamine method.

### 4.5. Chemical Cleavage of AP Site-Containing DNA

Under the chemical cleavage condition (200 mM NaOH, 37 °C for 15 min), AP site-containing DNA was cleaved at the AP site [59]. The cleavage products were separated by 20% denaturing polyacrylamide gel electrophoresis (PAGE) for 1 h, stained by GelRed and quantified.

### 4.6. Activity Analysis and Kinetic Assay

The substrate and the corresponding deoxyribozyme were first annealed (85 °C, 5 min; 4 °C, 5 min; 37 °C, 1 min); then the reaction was initiated by adding an equal volume of 2× reaction buffer (25 mM Mg^2+^, 50 mM HEPES, 200 mM NaCl, pH 7.5). The single turnover condition was 50 nM substrate and 500 nM deoxyribozyme. The reaction was stopped by adding two volumes of stop buffer (7 M urea, 1 mM EDTA, pH 8.0). The reaction products were separated by 20% denaturing PAGE for 1 h and quantified on a LI-COR Odyssey CLx imaging system using a 700 nm channel.

The observed reaction rate constant (*k*_obs_) was calculated using a pseudo-first-order reaction equation:

Y = Y_max_ [1 − exp(−*k*_obs_ × t)],

where Y is the percentage of the cleaved product at time t; Y_max_ is the percentage of cleaved product when the reaction reached a plateau (typically in the range of 91~99%). The *k*_obs_ values reported for deoxyribozyme-catalyzed RNA cleavage reactions represented the mean values of three independent experiments.

A Michaelis-Menten kinetics analysis was carried out under a multi-turnover reaction condition (10 nM enzyme and substrate concentration ranging from 100 to 5000 nM). Reported *k*_cat_ and *K*_M_ values were determined by the Michaelis-Menten equation:

v_obs_ = *k*_cat_ [E]/(*K*_M_ + [S]).

The observed reaction rate (v_obs_) for each substrate concentration ([S]) was calculated by a linear fit of at least three data points obtained over the first 5–25% of cleavage reaction.

### 4.7. Divalent Metal Ion Responsiveness of DNAzyme

Cleavage reactions are performed in 50 mM HEPES buffer (pH 7.5) containing 200 mM NaCl supplemented with 1 mM various divalent metal ions at 37 °C. After 50 nM substrate and 500 nM enzyme annealing, the reaction was initiated by adding an equal volume of buffer. The reaction was stopped using reaction stop buffer, and the results were analyzed with 20% PAGE.

### 4.8. In Vitro FRET Detection of miR-21

The MNAzyme substrate contained a fluorophore (FAM) and a quencher (BHQ-1) at its 5′ and 3′ end, respectively (Table S1). MiR-21 was annealed with Fragments 1 and 2 (200 nM) under 50 mM HEPES buffer (pH 7.5) containing 1 mM Mg^2+^ and 200 mM NaCl. The reaction was initiated by adding 400 nM MNAzyme substrate, which helps form the MNAzyme structure, resulting in cleavage of the substrate. Dissociation of cleavage products separated the fluorophore and quencher, leading to an increased fluorescence intensity. The MNAzyme’s fluorescence intensity was obtained on a SpectraMax ID3 microplate reader (excitation: 480 nm; emission: 520 nm) (Molecular Devices, San Jose, CA, USA).

For specificity of the proposed sensing MNAzyme for miRNA assays, the control experiments replaced miR-21 with single-base mismatch miRNA (3′-MT1, 5′-MT1), double-base mismatch miRNA (3′-MT2, 5′-MT2 and 5′/3′-MT2) and four other miRNAs (miRNA-29a, miRNA-155, miRNA-122 and miRNA-375). After 4 h of incubation, the fluorescence intensity was recorded. The limit of detection was examined under different concentrations of miR-21 (11Bn: 2 nM-2000 nM; WT: 10 nM-2000 nM). For the reduction experiments of MnO_2_ nanosheets, the reaction buffer was different by additionally adding the nanosheets and 0.2 mM GSH. The fluorescence intensity was recorded after 2 h of incubation.

### 4.9. Synthesis and Characterization of MnO_2_ Nanosheets

MnO_2_ nanosheets were synthesized according to a previous report. A mixture (20 mL) of TMA·OH (0.6 M) and H_2_O_2_ (3 wt%) was reacted with MnCl_2_·4H_2_O (10 mL, 0.3 M) within 15 s. After forming a dark brown suspension, the mixture was stirred overnight at room temperature, separated at 2000 rpm for 10 min, washed three times each with deionized water and methanol, and dried at 60 °C. Subsequently, 5 mg of the dried crude product was dispersed in 10 mL of deionized water and sonicated for 10 h. Finally, the supernate was used for experiments after centrifugation at 2000 rpm for 30 min [36].

TEM was performed using a JEOL transmission electron microscope operating at an accelerating voltage of 120 kV. Atomic force microscopy (AFM) images were obtained on Multimode 8 (Bruker, Billerica, MA, USA). The UV–vis absorption spectrum of MnO_2_ nanosheets was characterized by a Nanodrop 2000/2000C spectrophotometer (Thermo Fisher).

### 4.10. Cell Viability Assay

MDA-MB-231 cells were seeded into 96-well plates containing 100 μL of complete medium and incubated overnight. The medium was aspirated and 80 μL of complete medium was added again. The total concentration of NSs was 473 μg/mL calculated from the absorbance value and extinction coefficient. The concentration gradients were 0, 5, 10, 20, 30, 40, 50 and 60 μg/mL. In the case of 60 μg/mL, 12 μL of NSs was mixed with 8 μL of Opti-MEM medium (Gibco, Waltham, MA, USA) before being added to the wells. The reaction lasted for 24 h. Then 10 μL of Cell Counting Kit-8 was added to each well and incubated at 37 °C for 2 h. Finally, the absorbance was measured using a SpectraMax iD3 zymograph at 450 nm to determine the cell viability.

### 4.11. QPCR

Using the Total RNA Extraction Kit (Yeasen, Shanghai, China), 1 μg of total RNA was first mixed with 4× gDNA wiper mix and incubated at 42 °C for 2 min, followed by the addition of RT primer, RT Mix and Hiscript Enzyme Mix (25 °C, 5 min; 50 °C, 15 min; 85 °C, 5 min) to obtain cDNA. Subsequently, cDNA was analyzed by quantitative PCR (qPCR) using Taq Pro Universal SYBR qPCR Master Mix (Vazyme, Nanjing, China). Preparation system: 2 μL of cDNA template, 0.4 μL of miR-21 primer 1 and 2 and 10 μL of Master Mix. Subsequently, the PCR reaction was launched using the StepOnePlus instrument (Thermo Fisher, Waltham, MA, USA). After 40 rounds of PCR reaction, the Ct values of the three sets of replicate samples were measured and the relative copy number of miR-21 mRNA in each experimental group was calculated.

### 4.12. Intracellular Performance of miR-21 Sensor

MDA-MB-231, MCF10A and A549 cells (Cellcook, Guangzhou, China) were cultured in Dulbecco’s modified Eagle’s medium (DMEM) media supplemented with 10% fetal bovine serum (FBS), 100 U/mL penicillin and 100 μg/mL streptomycin solution (Gibco, Waltham, MA, USA) at 37 °C in a humidified 5% CO_2_ environment. Cells were subcultured every day or every other day. Before administration, cells were passed into 35 mm glass-bottomed culture dishes (MstTek Corp, Ashland, MA, USA) and allowed to grow for 24 h to reach 60–70% confluency.

The MNAzyme and/without MnO_2_ nanosheets (100 μL) were incubated with 900 μL of Opti-MEM at room temperature for 10 min and added to the living cells for 4 h at 37 °C. After that, cells were stained with Hoechst 33258 at a final concentration of 2.5 ng/mL at 37 °C for 8 min. PBS (0.1 M, pH 7.4) was used to wash cells three times, and fresh medium containing 10% fetal bovine serum was added. Then, cells were visualized under an Olympus laser scanning confocal microscope at 60× magnification. The fluorescence emission of Hoechst 33258 was measured over the 430–470 nm range, with excitation at 455 nm. Fluorescence of FAM was obtained by excitation at 517 nm and collected over 500–600 nm.

For flow cytometry analysis, after the same treatment as described for confocal imaging, cells were harvested, washed and resuspended in PBS. The samples were then sent for flow cytometry detection. Fluorescence intensity was measured using a flow cytometer (FITC channel). Data were analyzed using FlowJo software (v10.10.0). All experiments were performed in triplicate.

### 4.13. Transfection of miR-21 Inhibitor

The miRNA-21 inhibitor was transfected into MDA-MB-231 cells with Mirus TranslT at final amounts of 100 pmol, 200 pmol and 400 pmol according to the instructions. To be specific, Mirus TranslT (4 μL) and miRNA-21 inhibitor were incubated separately in 100 μL of Opti-MEM at room temperature for 10 min, then mixed and added to cells and incubated for 24 h. MDA-MB-231 cells were then incubated with fresh Opti-MEM medium containing the 11Bn/WT-based MNAzyme and MnO_2_ nanosheets for another 4 h, followed by staining with Hoechst 33258 and washing cells with PBS, and then we obtained fluorescent images on the confocal scanning system.

## 5. Conclusions

In summary, we report a more active DNAzyme variant, 11Bn, which exhibits up to 7-fold higher catalytic activity than the wild type. We constructed an 11Bn-based sensor for sensitive imaging of miR-21 in living cells. The sensor offers distinct advantages, including ease of preparation and operation, as well as improved detection sensitivity without signal amplification. This work provides a versatile molecular tool for low-abundance RNA imaging and holds promise for early disease diagnosis.

## 6. Patents

H.Y., J.C., Z.Z. and Z.L. have filed a patent regarding the preparation of chemically modified DNAzyme sensors and the use of chemically modified sensors for imaging nucleic acids in living cells.

## Data Availability

The data presented in this study are available within the article and Supplementary Materials.

## Supplementary Materials

The following supporting information can be downloaded at: https://www.mdpi.com/article/10.3390/molecules31081271/s1, Figure S1: Gel electrophoresis analysis of the reaction products at different stages; Figure S2: The catalytic analysis of DNAzyme 8-17 variants with site-specific chemical modifications; Figure S3: Dual-site chemical modification on DNAzyme 8-17; Figure S4: *k*_obs_ values of 11Bn- or BnNb-catalyzed chimeric substrate cleavage; Figure S5: Mass spectra of WT DNAzyme 8-17; Figure S6: Michaelis-Menten curve for 11Bn-catalyzed reaction; Figure S7: Time-dependent fluorescence intensity of MNAzyme constructed by WT/11Bn DNAzymes with short binding arms; Figure S8: Enzyme:substrate concentration ratio affects MNAzyme’s catalytic activity; Figure S9: Multi-turnover activity of 11Bn MNAzyme; Figure S10: Reduction of MnO_2_ NS by GSH; Figure S11: Standard curve showing the linear range of MnO_2_ NS absorbance versus concentration; Figure S12: MnO_2_ NS facilitates cellular delivery of miR-21 sensors; Figure S13: Flow cytometry analysis of MDA-MB-231 cells; Figure S14: Sensitive imaging of intracellular miRNA in A549 cells; Figure S15: Flow cytometry analysis of A549 cells; Figure S16: Quantification of relative miR-21 expression levels; Table S1: Sequences used in this study; Table S2: *k*_obs_ values of WT- or 11Bn-catalyzed cleavage reactions.

## Abbreviations

The following abbreviations are used in this manuscript:

UV–vis	Ultraviolet–visible spectroscopy
QPCR	Quantitative real-time polymerase chain reaction
