# Supplementary material for: Chemically Modified DNAzyme with Enhanced Activity for Sensitive MicroRNA Imaging in Live Cells
Source: Molecules. 2026 Apr 12;31(8):1271. doi: 10.3390/molecules31081271 (PMC13119030; doi:10.3390/molecules31081271)
Supplement: Supplementary file 1 [file molecules-31-01271-s001.zip › molecules-4222399-supplementary.pdf]

Supplementary information

Chemically Modified DNAzyme with Enhanced Activity for Sensitive MicroRNA Imaging in Live Cells

Jiawen Chen [1], Juan Wang [2], Jiahuan Wang [1], Fulong Wang [1], Wenyu Cheng [1], Siqi Chen [1], Rui Mo [1] and Hanyang Yu [1,*]

[1] State Key Laboratory of Coordination Chemistry, College of Engineering and Applied Sciences, Chemistry and Biomedicine Innovation Center (ChemBIC), Nanjing University, Nanjing 210023, China; 502023340052@smail.nju.edu.cn (J.C.); wjhuan_27@163.com (J.W.); fulongwang0908@foxmail.com (F.W.); chengwenyu16@gmail.com (W.C.); siqichen0812@163.com (S.C.); 652023340040@smail.nju.edu.cn (R.M.)

[2] State Key Laboratory of Coordination Chemistry, School of Chemistry and Chemical Engineering,
Chemistry and Biomedicine Innovation Center (ChemBIC), ChemBioMed Interdisciplinary Research Center, Nanjing University, Nanjing 210023, China; dg20240105@smail.nju.edu.cn

* Correspondence: hanyangyu@nju.edu.cn

Contents

Supplementary Figures S1-S16...S3-S18
Supplementary Tables S1-S2...S19-S20

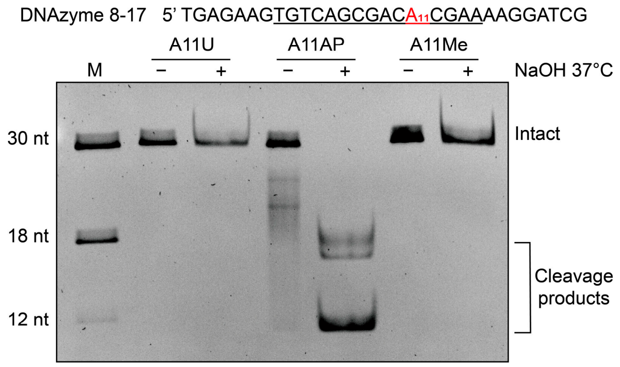

Figure S1. Gel electrophoresis analysis of the reaction products at different stages. Variant of DNAzyme 8-17 with a methyl modification site-specifically installed at position 11 (in red) within the catalytic core region (underlined) was prepared. Oligonucleotide with an abasic (AP) site is susceptible to cleavage under alkaline condition (200 mM NaOH, 37 °C, 15 min). The resulting A11AP underwent efficient cleavage via α, β elimination, along with incomplete γ, δ elimination under same conditions, producing three fragments: a 5' fragment with a sugar ring (18 nt), a 5' fragment lacking the sugar ring but containing a 3'-phosphate, and a 3' fragment with a 5'-phosphate (12 nt). The partial degradation of the untreated band was likely caused by elevated temperature during gel electrophoresis. M: ssDNA marker.

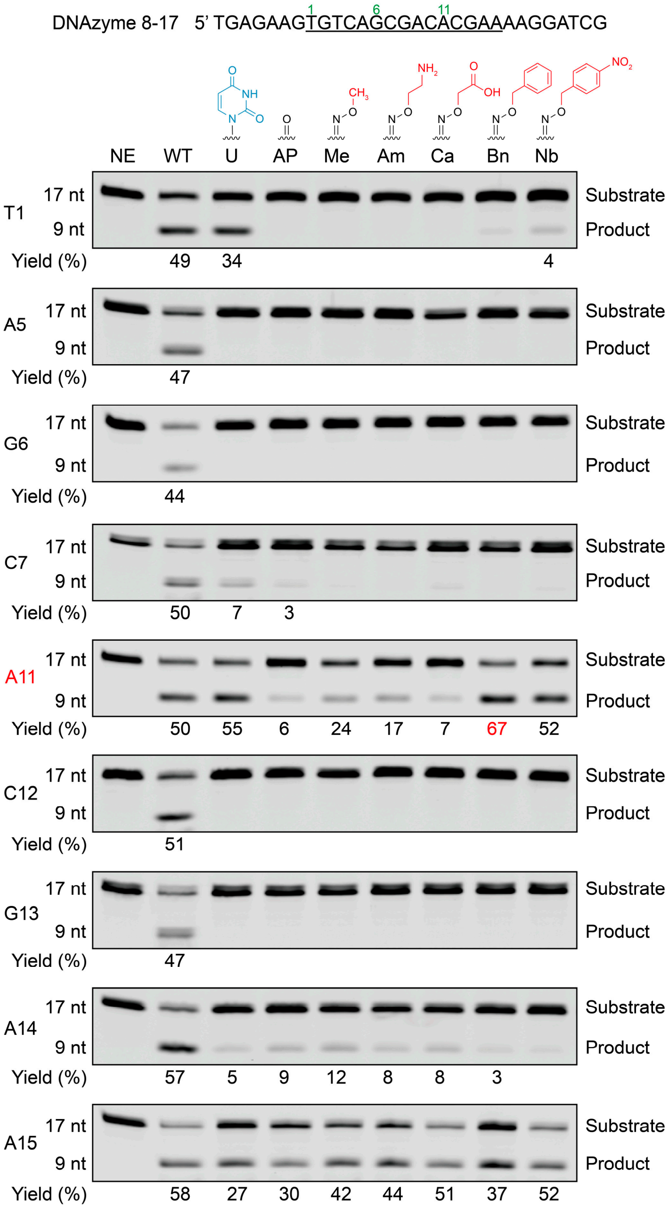

Figure S2. The catalytic analysis of DNAzyme 8-17 variants with site-specific chemical modifications. The catalytic core region is underlined. Cleavage reactions were performed in 50 mM HEPES buffer (pH 7.5) containing 25 mM Mg^{2+} and 200 mM NaCl at 37 °C for 30 sec. [Enzyme] = 500 nM. [Substrate] = 50 nM.

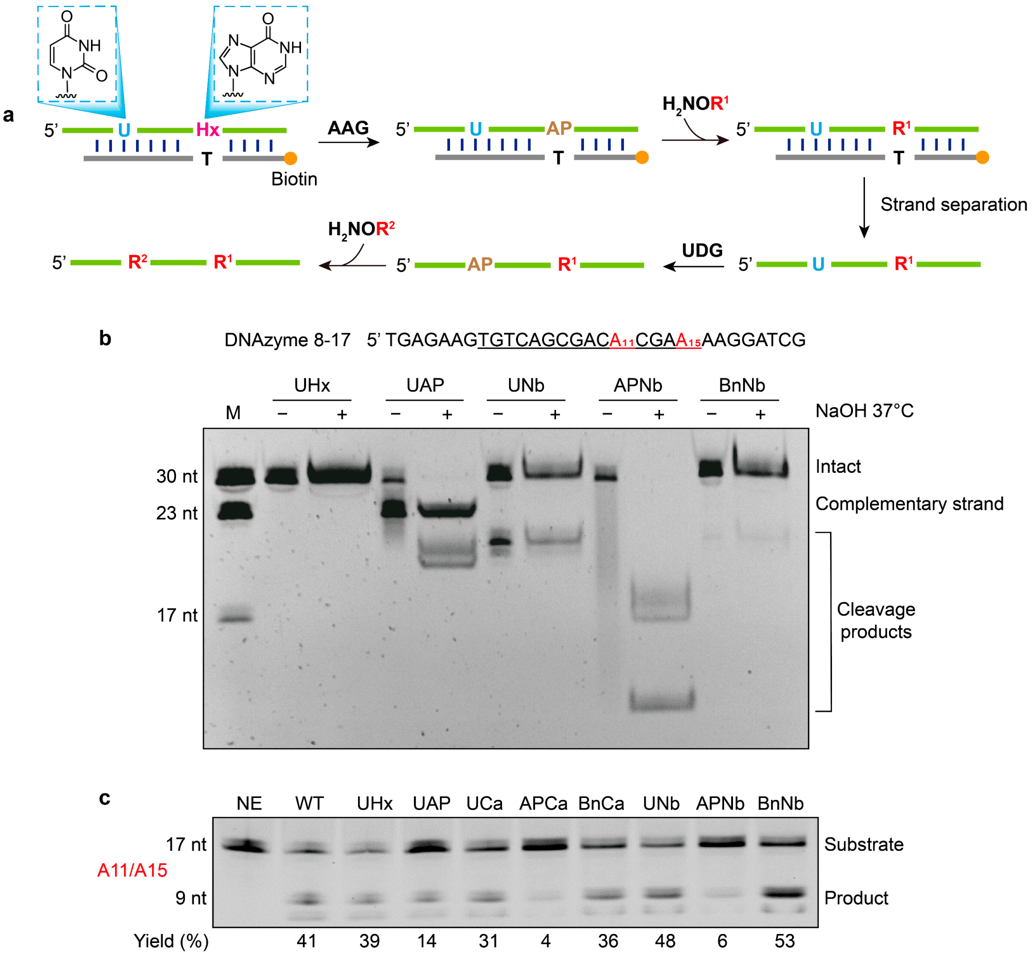

Figure S3. Dual-site chemical modification on DNAzyme 8-17. (a) Scheme of sequential installation of two modifications onto DNA. DNAzyme 8-17 with two noncanonical bases (U, uracil; Hx, hypoxanthine) annealed to a biotinylated complementary sequence is first treated with AAG (200 U/mL, 37 °C, 4 h) to excise Hx and generate an AP site. The AP site-containing DNA is incubated with an oxyamine compound to introduce the first functional group (R^1). The second functional group (R^2) is introduced by the UDG-oxyamine method. (b) Gel electrophoresis analysis of the reaction products at different stages. Variant of DNAzyme 8-17 with a benzyl modification and a carboxyl modification installed at position 11 and position 15 (in red), respectively, within the catalytic core region (underlined) was prepared. After alkaline treatment (200 mM NaOH, 37 °C, 15 min), the variant APNb produced bands corresponding to a 5' fragment with a 5' sugar ring, a 5' fragment lacking the sugar ring, and a 3' fragment with a 5'-phosphate, whereas BnNb withstood the chemical cleavage conditions. (c) The catalytic analysis of DNAzyme 8-17 variants with dual-site chemical modifications on position 11 and 15. Cleavage reactions were performed in 50 mM HEPES buffer (pH 7.5) containing 25 mM Mg^{2+} and 200 mM NaCl at 37 °C for 30 sec. [Enzyme] = 500 nM. [Substrate] = 50 nM.

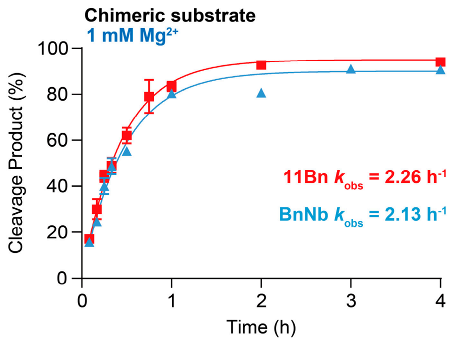

Figure S4. k_{obs} values of 11Bn- or BnNb- catalyzed chimeric substrate cleavage. Cleavage reactions were performed in 50 mM HEPES buffer (pH 7.5) containing 200 mM NaCl and 1 mM Mg^{2+} at 37 °C. [Enzyme] = 500 nM. [Substrate] = 50 nM. The error bars denote ± S.D. of the mean for n = 3 independent replicates.

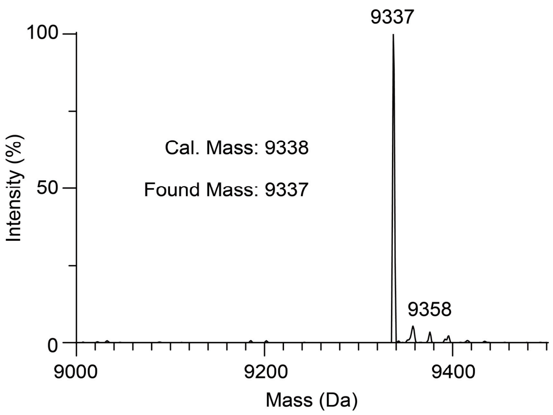

Figure S5. Mass spectra of WT DNAzyme 8-17.

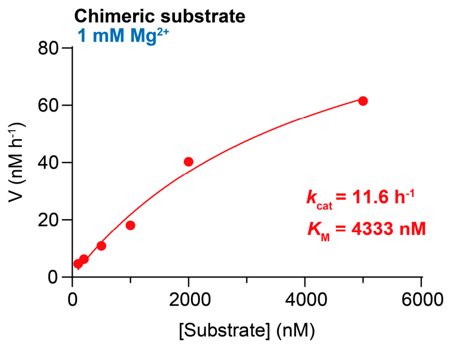

Figure S6. Michaelis-Menten curve for 11Bn-catalyzed reaction. K_M and k_{cat} were determined under a multiple turnover condition (10 nM enzyme; chimeric substrate concentration ranging from 100 to 5,000 nM). Cleavage reactions were performed in 50 mM HEPES buffer (pH 7.5) containing 1 mM Mg^{2+} and 200 mM NaCl at 37 °C. The error bars denote ± S.D. of the mean for $n = 3$ independent replicates.

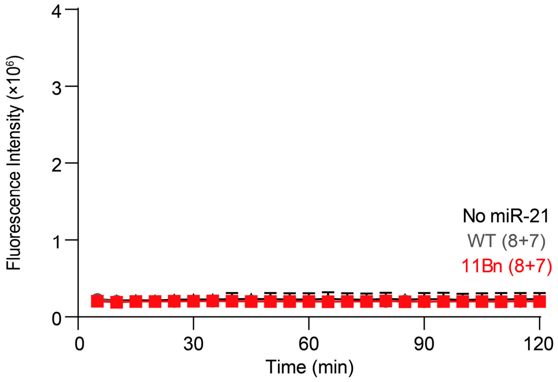

Figure S7. Time-dependent fluorescence intensity of MNAzyme constructed by WT/11Bn DNAzymes with short binding arms (8 nt + 7 nt). The reactions were performed in 50 mM HEPES buffer (pH 7.5) containing 10 mM Mg^{2+} and 200 mM NaCl at 37 °C for 2 h. [Enzyme] = 200 nM. [Substrate] = 200 nM. Error bars denote ± S.D. of the mean for $n = 3$ independent replicates.

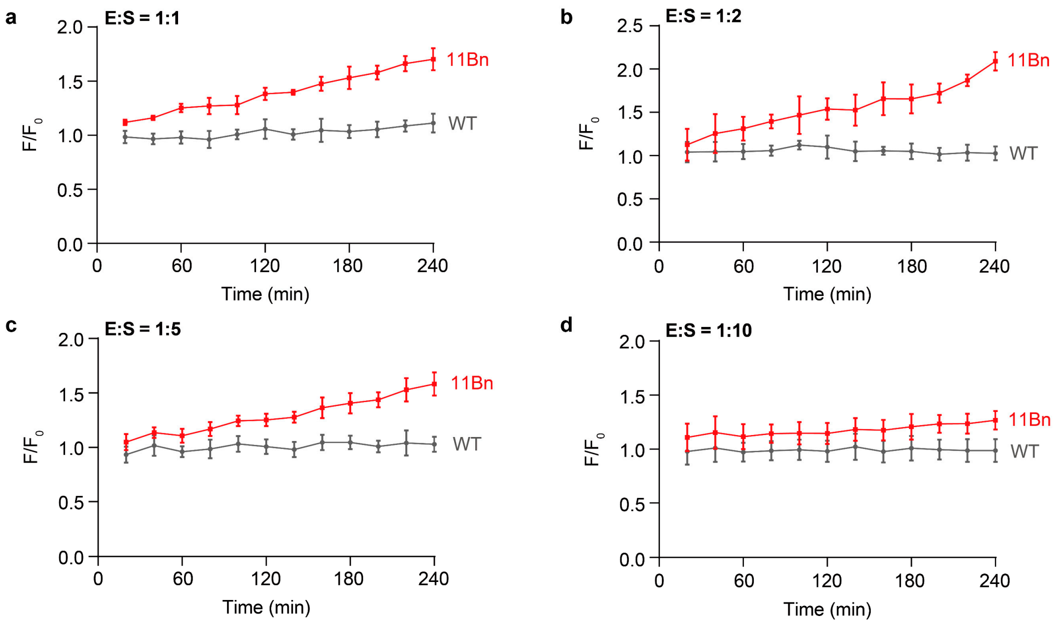

Figure S8. Enzyme:substrate concentration ratio affects MNAzyme's catalytic activity. Fluorescence intensity of 11Bn/WT-based MNAzyme at different enzyme:substrate concentration ratios. The reactions were performed in 50 mM HEPES buffer (pH 7.5) containing 1 mM Mg^{2+} and 200 mM NaCl at 37 °C for 4 h. [Enzyme] = 200 nM; [Substrate] = (a) 200 nM, (b) 400 nM, (c) 1000 nM, (d) 2,000 nM. F and F_0 represent sensor fluorescence intensities in the presence and absence of miR-21, respectively. Error bars denote ± S.D. of the mean for n = 3 independent replicates.

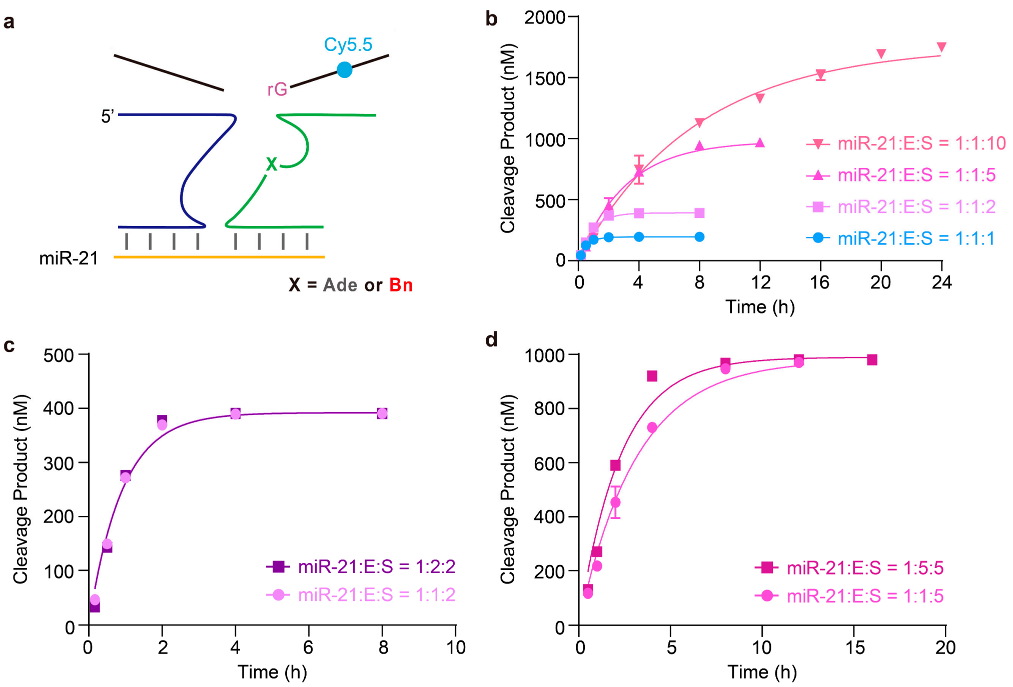

Figure S9. Multi-turnover activity of 11Bn MNAzyme. (a) Scheme of MNAzyme-catalyzed multiple turnover of Cy5.5-labeled substrate. (b) Multi-turnover activity of MNAzyme. Substrate concentration ranges from 200 to 2,000 nM. (c-d) Impact of DNAzyme concentration on reaction kinetics under fixed substrate concentrations. [Substrate] = (c) 400 nM, (d) 1000 nM. [miR-21] = 200 nM. Cleavage reactions were performed in 50 mM HEPES buffer (pH 7.5) containing 200 mM NaCl and 10 mM Mg^{2+} at 37 °C. The error bars denote ± S.D. of the mean for n = 3 independent replicates.

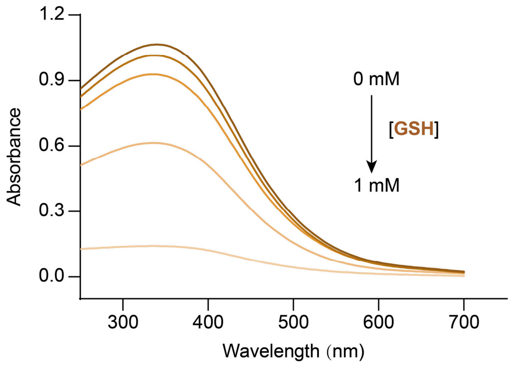

Figure S10. Reduction of MnO_2 NS by GSH. UV-vis absorption spectrum of MnO_2 NS after treated with GSH of different concentrations ranging from 0 mM to 1 mM (from top to bottom: 0 mM, 0.1 mM, 0.5 mM, 0.75 mM, 1 mM).

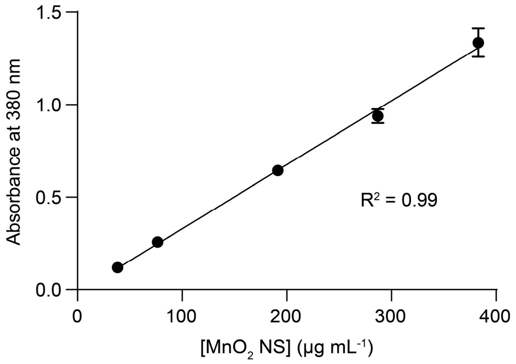

Figure S11. Standard curve showing the linear range of MnO_2 NS absorbance versus concentration.

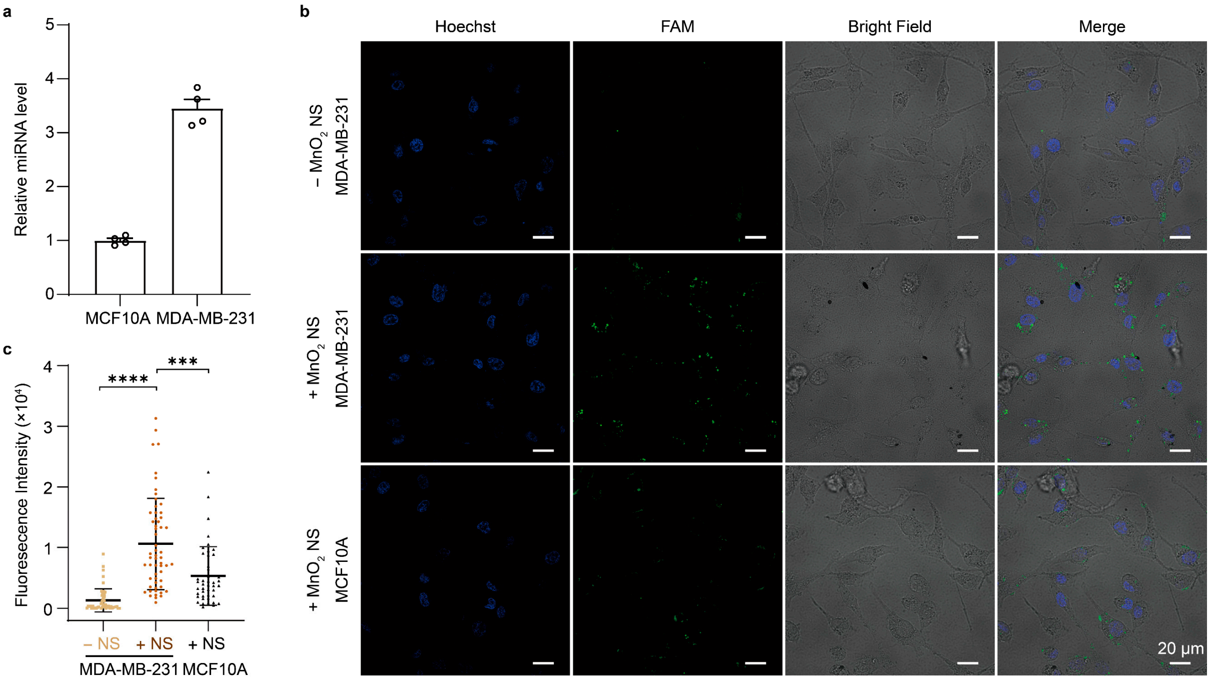

Figure S12. MnO_2 NS facilitates cellular delivery of miR-21 sensors. (a) Quantification of relative miR-21 expression levels in MCF10A and MDA-MB-231. Error bars denote ± S.D. of the mean for n = 4 independent replicates. (b) Confocal fluorescence images and (c) quantification of MDA-MB-231 and MCF10A cells after incubation with 11Bn-based MNAzymes with or without MnO_2 NS (20 μg/mL) for 6 h. Statistical analysis was performed using a two-tailed t test. ***: $p < 0.001$. ****: $p < 0.0001$. Data represent mean ± S.D. (n = 49, 57 and 44 cells for -NS MDA-MB-231, +NS MDA-MB-231 and +NS MCF10A, with fluorescence intensity of 1.3×10^3, 1.1×10^4 and 5.3×10^3 respectively).

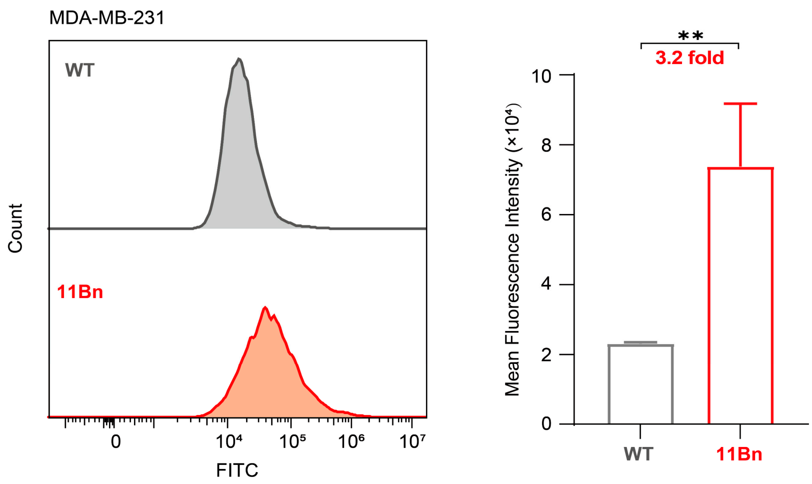

Figure S13. Flow cytometry analysis of MDA-MB-231 cells after incubation with WT- or 11Bn-based MNAzymes and MnO_2 NS (33 μg/mL) for 4 h. **: $p < 0.01$. The error bars denote ± S.D. of the mean for $n = 3$ independent replicates.

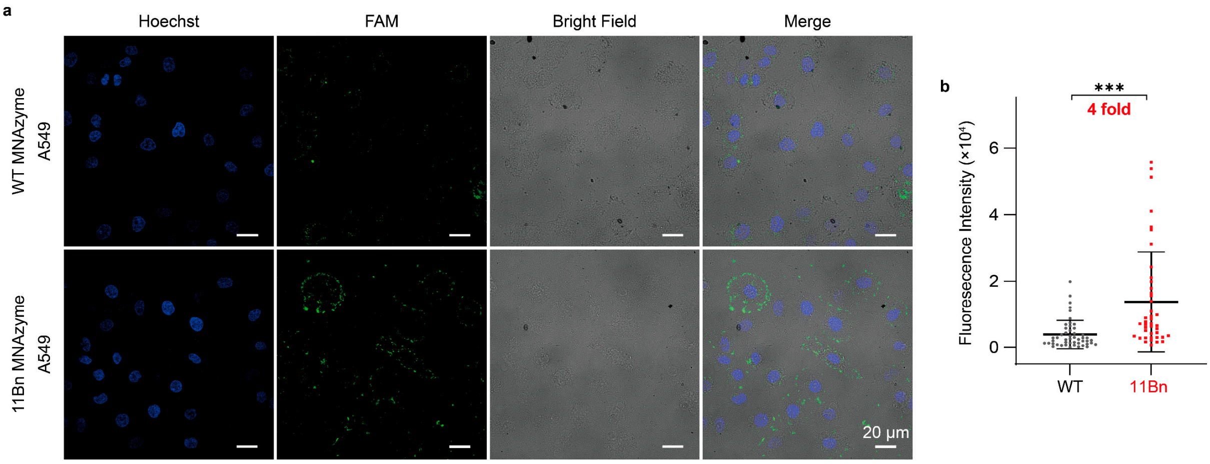

Figure S14. Sensitive imaging of intracellular miRNA in A549 cells. (a) Confocal fluorescence images and (b) quantification of A549 cells after incubation with WT- or 11Bn-based MNAzymes and MnO_2 NS for 4 h. Statistical analysis was performed using a two-tailed t test. ***: $p < 0.001$. Data represent mean $\pm$ S.D. (n = 49 and 43 cells for WT-A549 and 11Bn-A549, with fluorescence intensity of 3.9×10^3 and 1.7×10^4 respectively).

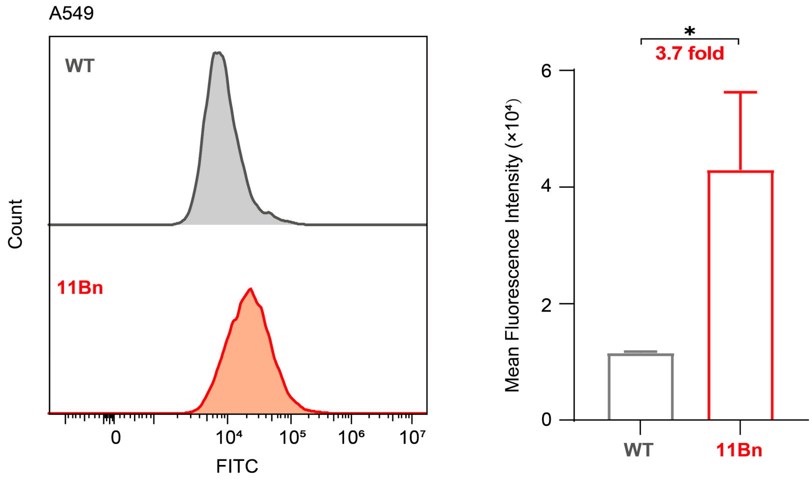

Figure S15. Flow cytometry analysis of A549 cells after incubation with WT- or 11Bn-based MNAzymes and MnO_2 NS (33 μg/mL) for 4 h. *: $p < 0.05$. The error bars denote ± S.D. of the mean for $n = 3$ independent replicates.

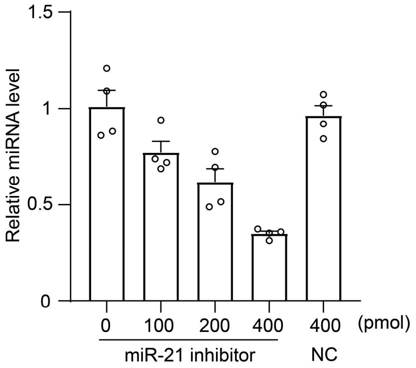

Figure S16. Quantification of relative miR-21 expression levels in MDA-MB-231 cells after different amounts of miR-21 inhibitor treatment. Error bars denote ± S.D. of the mean for *n* = 4 independent replicates.

Table S1. Sequences used in this study. Black: DNA. Red: noncanonical base in DNA. Blue: RNA. Orange: labeled base in DNA. Green: 2'-O-methylated RNA. U: uracil. Hx: hypoxanthine. Catalytic core region is underlined.

**Oligo Name**	**Sequence (5' to 3')**
DNAzyme 8-17 WT	TGAGAAGTGTCAGCGACACGAAAAGGATCG
T1U	TGAGAAGUGTCAGCGACACGAAAAGGATCG
A5U	TGAGAAGTGTCUGCGACACGAAAAGGATCG
G6U	TGAGAAGTGTCAUCGACACGAAAAGGATCG
C7U	TGAGAAGTGTCAGUGACACGAAAAGGATCG
A11U	TGAGAAGTGTCAGCGACUCGAAAAGGATCG
C12U	TGAGAAGTGTCAGCGACAUGAAAAGGATCG
G13U	TGAGAAGTGTCAGCGACACUAAAAGGATCG
A14U	TGAGAAGTGTCAGCGACACGUAAAGGATCG
A15U	TGAGAAGTGTCAGCGACACGAUAAGGATCG
A11U/A15Hx	TGAGAAGTGTCAGCGACUCGAHxAAGGATCG
Complement	Biotin-CGATCCTTTTCGAGTCGCTGACA
Fragment 1 (8+7)	TCAACATCAGTGACACGAAAAGGATCG
Fragment 1U (8+7)	TCAACATCAGTGACUCGAAAAGGATCG
Fragment 2 (8+7)	TGAGAAGTGTCAGCCTGATAAGCTA
Fragment 1 (11+11)	TCAACATCAGTGACACGAAAAGGATCGTCT
Fragment 1U (11+11)	TCAACATCAGTGACUCGAAAAGGATCGTCT
Fragment 2 (11+11)	CGCATGAGAAGTGTCAGCCTGATAAGCTA
**Substrate**	
Chimeric S	Cy5.5-CGATCCTTGGCTTCTCA
All-RNA S	Cy5.5-CGATCCTTGGCTTCTCA
S (8+7)	CGAT(FAM)CCTTGGCTTCT(BHQ1)CA
S (11+11)	AGACGATCCT(FAM)TGGCTT(BHQ1)CTCATGCG
S (11+11) (Cy5.5)	Cy5.5-AGACGATCCTTGGCTTCTCATGCG
**Others**	
MiR-21	UAGCUUAUCAGACUGAUGUUGA
3'-MT1 miR-21	UAGCUUAUCAGACUGAUGAUGA
5'-MT1 miR-21	UAGGUUAUCAGACUGAUGUUGA
3'-MT2 miR-21	UAGCUUAUCAGACUGAUGAUCA
5'-MT2 miR-21	UUGGUUAUCAGACUGAUGUUGA
5'/3'-MT2 miR-21	UUGCUUAUCAGACUGAUGUUCA
MiR-155	UUAAUGCUAAUCGUGAUAGGGGU
MiR-122	UGGAGUGUGACAAUGGUGUUUG
MiR-375	UUUGUUCGUUCGGCUCGCGUGA
MiR-21 inhibitor	UCAACAUCAGUCUGAUAAGCUA
Forward Primer	GCGCGTAGCTTATCAGACTGA
Reverse Primer	ATCCAGTGCAGGGTCCGAGG
RT Primer	GTCGTATCCAGTGCAGGGTCCGAGGTATTCGCACTGGATACGACTCAACA

Table S2. k_{obs} values of WT- or 11Bn-catalyzed cleavage reactions. [Enzyme] = 500 nM. [Substrate] = 50 nM.

Substrate	Metal ion cofactor		k_{obs} (h^{-1})	
	Mn^{2+}	Mg^{2+}	11Bn	WT
Chimeric substrate	10 µM	0	1.26	0.17
	0	0.1 mM	0.37	0.09
	0	1 mM	2.26	0.67
	0	25 mM	165	90
	10 µM	1 mM	6.45	1.44
All-RNA substrate	1 mM	0	0.53	0.13
	0	10 mM	0.43	0.11
	1 mM	10 mM	7.86	1.68
